# Complete sequence of mitochondrial DNA of red alga dulse *Palmaria palmata* (Linnaeus) Weber & Mohr in Japan

**DOI:** 10.1080/23802359.2019.1668733

**Published:** 2019-09-24

**Authors:** Yuya Kumagai, Ryota Tsubouchi, Yoshikatsu Miyabe, Tomoyuki Takeda, Kohsuke Adachi, Hajime Yasui, Hideki Kishimura

**Affiliations:** a Laboratory of Marine Chemical Resource Development, Faculty of Fisheries Sciences, Hokkaido University, Hakodate, Japan;; b Chair of Marine Chemical Resource Development, Graduate School of Fisheries Sciences, Hokkaido University, Hakodate, Japan;; c Laboratory of Aquatic Product Utilization, Graduate School of Agriculture, Kochi University, Nankoku, Japan;; d Laboratory of Humans and the Ocean, Faculty of Fisheries Sciences, Hokkaido University, Hakodate, Japan

**Keywords:** Mitochondrial genome, macroalgae, Pacific dulse, red alga

## Abstract

Red algae contain high amount of proteins compared to the other algae. Red algae dulse is one of the protein rich species and a good candidate for protein sources. In this study, the complete mitochondrial genome of *Palmaria palmata* in Japan was determined. It had a circular mapping molecular with the length of 31,399 bp and contained 53 genes including 27 protein-coding, 2 rRNA, and 24 tRNA. Phylogenetic analysis showed that *Palmaria palmata* in Japan was separated with Atlantic dulse. This is the first report of complete mitochondrial genome from Pacific dulse.

Marine algae contain valuable nutrition such as proteins, lipids, carbohydrates, vitamin and minerals. Among them, red algae contain a high amount of protein (7–30% of dry weight) (Holdt and Kraan 2011), and plastid proteins are related to the amount of total protein in seaweeds, especially phycobiliproteins and ribulose-1,5-bisphosphate carboxylase/oxygenase. Many bioactive peptides were obtained from the hydrolysates such as inhibition of angiotensin I converting enzyme (ACE) (Kitade et al. 2018) and dipeptidyl peptidase IV (Harnedy et al. 2015), anti-diabetic (Harnedy and FitzGerald 2013), antioxidant (Sato et al. 2019) and cell proliferation (Yuan et al. 2005). ACE inhibitory peptides were also reported in Pacific and Atlantic dulse (Fitzgerald et al. 2012; Harnedy et al. 2014; Furuta et al. 2016; Miyabe et al. 2017). We compared the potential ACE inhibitory peptides from plastid protein sequences between Pacific and Atlantic dulse, revealing that the amounts of bioactive peptides and peptide structures differed (Kumagai et al. 2019). To clarify the characteristics of protein rich red algae dulse, we determined the complete mitochondrial DNA of *Palmaria palmata* in Japan.

DNA was extracted from *P*. *palmata* in Japan (Specimen Voucher HUF 20120202001) collected from the intertidal at Usujiri, Japan (N 41.936002, E 140.950406) by the CTAB method (Cota-Sánchez et al. 2006). The library construction and sequencing were performed by the GS Junior Titanium Series system (Roche). The data were assembled using default de novo settings in CLC Genomics Workbench 8.5.1 and annotated following Watanabe et al. (2019). Phylogenetic analyses were performed using amino acid sequences of *cox1*, *psbA* and *rbcL*. The best-fit model for maximum likelihood was GTR + I + G and analysed using MEGA X. Bootstrap probability values were run with 1000 replicates. *Rhodothamniella floridula* was used as outgroup.

The complete mitogenome of *P*. *palmata* in Japan comprised a circular DNA molecule with the length of 31,399 bp (DDBJ accession No. AP019296). The overall GC content of the complete mitogenome was 32.2%. The mitogenome contained 53 genes, including 27 protein-coding, two rRNA, 24 tRNA genes. Of the 27 protein-coding genes, 19 were terminated with TAA stop codon, except for *rpl20*, *nad2*, *nad3*, *nad4*, *nad4L*, *nad6*, *atp9* and *tatC* with TAG. Two introns were detected in *trnI* (494 bp) and ribosomal RNA large subunit (*rrl*) (2,463 bp). BLASTX analysis revealed that the *rrl* intron showed the similarity to that of the hypothetical protein from *Ahnfeltia plicata* (75%) and *orf544* from *Porphyra purpurea* (61%), which were suggested as a group II intron reverse transcriptase/maturase (Burger et al. 1999), however, the gene in *P*. *palmata* in Japan was incomplete because of a frameshift. Phylogenetic analysis showed that *t*he tree showed the same branch as previous report (Saunders et al. 2018), and *P*. *palmata* in Japan was separated from Atlantic dulse and included in the branch of *Palmaria mollis* (Figure 1).

**Figure 1.**
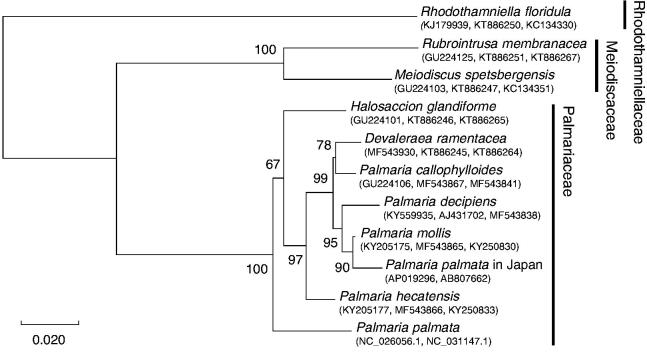
Maximum likelihood (ML) phylogeny generated from the three-gene alignment (*cox1*+*psbA*+*rbcL*) with ML bootstrap support values. The scale bar indicates substitutions per site.
